# A Community-Based, Case-Control Study Evaluating Mortality Reduction from Gastric Cancer by Endoscopic Screening in Japan

**DOI:** 10.1371/journal.pone.0079088

**Published:** 2013-11-13

**Authors:** Chisato Hamashima, Kazuei Ogoshi, Mikizo Okamoto, Michiko Shabana, Takuji Kishimoto, Akira Fukao

**Affiliations:** 1 Cancer Screening Assessment and Management Division, Research Center for Cancer Prevention and Screening, National Cancer Center, Tokyo, Japan; 2 Niigata Cancer Center Hospital, Niigata, Japan; 3 Division of Health Administration and Promotion, Department of Social Medicine, Faculty of Medicine, Tottori University, Yonago, Tottori, Japan; 4 Department of Internal Medicine, San-in Rosai Hospital, Yonago, Tottori, Japan; 5 Division of Environmental and Preventive Medicine, Department of Social Medicine, Faculty of Medicine, Tottori University, Yonago, Tottori, Japan; 6 Departments of Public Health, School of Medicine, Yamagata University, Yamagata, Yamagata, Japan; University of Texas MD Anderson Cancer Center, United States of America

## Abstract

**Aims:**

Although the incidence of gastric cancer has decreased in the last 3 decades, it remains the second leading cause of cancer death worldwide. In Asian countries, the burden of gastric cancer has remained, and cancer screening is normally expected to reduce gastric cancer death. We conducted a community-based, case-control study to evaluate the reduction of mortality from gastric cancer by endoscopic screening.

**Methods:**

Case subjects were defined as individuals who had died of gastric cancer between 2003 and 2006 in 4 cities in Tottori Prefecture, and between 2006 and 2010 in Niigata City, Japan. Up to 6 control subjects were matched by sex, birth year (±3 years), and the residence of each corresponding case subject from the population lists in the study areas. Control subjects were required to be disease-free at the time when the corresponding case subjects were diagnosed as having gastric cancer. The odds ratios (ORs) were calculated for those who had participated in endoscopic or radiographic screening before the reference date when the case subjects were diagnosed as having gastric cancer, compared with subjects who had never participated in any screening. Conditional logistic-regression models for matched sets were used to estimate the ORs and 95% confidence intervals (CIs).

**Results:**

The case subjects consisted of 288 men and 122 women for case subjects, with 2,292 matched control subjects. Compared with those who had never been screened before the date of diagnosis of gastric cancer in the case subjects, the ORs within 36 months from the date of diagnosis were 0.695 (95% CI: 0.489–0.986) for endoscopic screening and 0.865 (95% CI : 0.631–1.185) for radiographic screening.

**Conclusions:**

The results suggest a 30% reduction in gastric cancer mortality by endoscopic screening compared with no screening within 36 months before the date of diagnosis of gastric cancer.

## Introduction

Although the incidence of gastric cancer has decreased in the last 3 decades, it remains the second leading cause of cancer death worldwide [1]. The highest mortality rates are estimated in Eastern Asia, including Japan. The incidence of gastric cancer in the world was estimated to be about 1 million in 2008, half of which occurred in Eastern Asia.

In Asian countries, the burden of gastric cancer has persisted; however, there is as yet no nationwide population-based screening for gastric cancer except in Korea and Japan [2]. In Japan, there were 49,830 recorded deaths from gastric cancer in 2011, accounting for 13.9% of all cancer deaths [3]. Gastric cancer screening using upper gastrointestinal series (i.e., radiographic screening), which was developed in Japan, has been conducted for the last 3 decades as a public policy and its inclusion was recommended in the Japanese guidelines for gastric cancer screening [4]. It is expected that cancer screening will continue to prevent gastric cancer deaths in Japan, wherein 3.7 million Japanese participated in gastric cancer screening in 2010 [5].

Endoscopy has been commonly used in clinical practice and is anticipated to be an alternative strategy to radiography for gastric cancer screening. In Korea, endoscopic screening as a national program has been conducted since 2000 [6]. This has been partly adopted in population-based screening in Japan [7]–[9]. Although positive results of endoscopic screening have been reported recently, the effectiveness of endoscopic screening remains unclear [7]–[8], [10]–[12]. To effectively introduce endoscopy as a new method for gastric cancer screening in a community, mortality reduction from gastric cancer must be evaluated by conducting reliable studies. Although a randomized controlled trial is ideal for clarifying such reduction, case-control studies are useful for evaluating the effectiveness of cancer screening on a widespread scale [13]. To evaluate reduction of mortality from gastric cancer by endoscopic screening, we conducted a community-based, case-control study in Tottori and Niigata Prefectures, Japan, where endoscopic screening for gastric cancer has been conducted.

## Methods

### Study Population

Five cities (i.e., Tottori, Yonago, Kurayoshi and Sakaiminato in Tottori Prefecture and Niigata in Niigata Prefecture) that conducted endoscopic screening for at least 5 years and have local cancer registries were selected [7], [9]. These cities had higher mortality rates for gastric cancer than the other cities in Japan (16.2 per 100,000 individuals for men; 6.1 per 100,000 individuals for women) [3]. The age-adjusted mortality rates per 100,000 individuals up to 75 years of age were 20.3 for men and 6.9 for women in Tottori Prefecture and 20.3 for men and 6.0 for women in Niigata Prefecture.

### Screening Programs

Similar systems for gastric cancer screening are offered in these 5 cities [7], [9]. Gastric cancer screening is offered annually by local governments, and both radiography and endoscopy are used in these cities. All individuals aged 40 years and over can participate in the screening programs for gastric cancer. Endoscopic screening has been conducted since 2000 in Tottori, Yonago, and Sakaiminato, since 2001 in Kurayoshi, and since 2003 in Niigata. Individuals can choose either endoscopy or radiography for gastric cancer screening based on their preference. Participation in gastric cancer screening has increased since the introduction of endoscopic screening, but the participation in gastric cancer screening involving both methods has remained at about 25% [7], [9].

Physicians who can perform endoscopic screening were approved by the local committee for gastric cancer screening based on certain requirements [7], [9]. Although endoscopic screening has been performed in clinical settings, the results have been evaluated based on monitor screen review by the local committee, including experienced endoscopists in each city.

### Selection of Case Subjects

**Figure 1:**
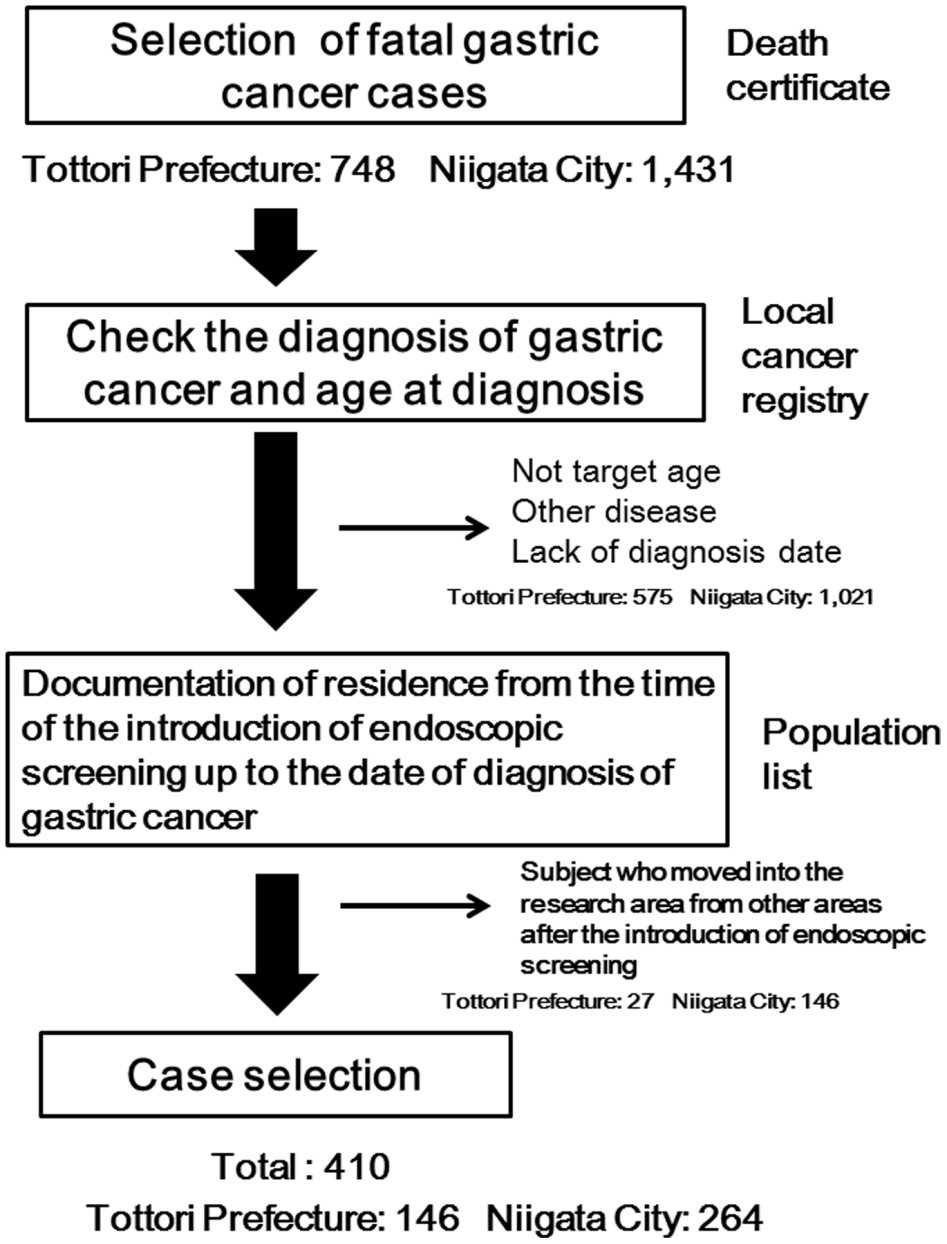
Flowchart for the selection of case subjects. Case subjects were defined as individuals who had died of gastric cancer from January 2003 to December 2006) were over 80 years old and less than 39 years old at the time of diagnosis, 2) lacked the date of gastric cancer diagnosis, or 3) had a diagnosis other than cancer. In the population list of each city, the remaining subjects were identified based on documentation of residence from the time of the introduction of endoscopic screening up to the date of diagnosis. There were1,769 subjects who were excluded because they did not fulfill the basic requirements for case subjects. The remaining 410 subjects (146 from Tottori Prefecture and 264 from Niigata City) were evaluated in the study.

The flowchart for the selection of case subjects is shown in **Figure 1**. Case subjects were defined as individuals who had died of gastric cancer from January 2003 to December 2006 in the 4 cities in Tottori Prefecture, and from April 2006 to October 2010 in Niigata City. Those who died of gastric cancer were identified by death certificates, with permission from the Japanese government. Case subjects were also diagnosed as having gastric cancer between January 2003 and December 2006 based on the Tottori Prefecture Cancer Registry and between April 2006 and October 2010 based on the Niigata Prefecture Cancer Registry. The age at diagnosis was limited between the ages of 40 years and 79 years. Individuals who died of malignant lymphoma and other gastric diseases were excluded. The case subjects had lived in the 5 cities from the date of the introduction of endoscopic screening up to the date of diagnosis of gastric cancer.

There were 748 potential subjects with gastric cancer in the 4 cities in Tottori Prefecture (326 in Tottori, 255 in Yonago, 112 in Kurayoshi, and 55 in Sakaiminato) and 1,431 potential subjects in Niigata City based on the death certificates. Detail information of all potential cases were obtained from the local cancer registries, and the following cases were excluded: patients who 1) were over 80 years old and less than 39 years old at the time of diagnosis, 2) lacked the date for gastric cancer diagnosis, or 3) had a diagnosis other than cancer. Most subjects who were excluded from the target group were over 80 years old at the time of diagnosis, which was not the actual target for cancer screening. In the population list of each city, the remaining patients were identified based on documentation of residence from the time of the introduction of endoscopic screening up to the date of gastric cancer diagnosis. There were 1,769 subjects who were excluded because they did not fulfill the basic requirements for case subjects, and the remaining 410 subjects (146 in Tottori Prefecture and 264 in Niigata City) were evaluated in this study.

### Selection of Control Subjects

Six control subjects were selected from the list of residents in each city for each matched case. The control subjects were required to be disease-free at the time when the case subjects were diagnosed as having gastric cancer. The control subjects were matched by sex, birth year (±3 years), and residence of the matched case subjects from the population lists. The population lists at the time of the introduction of endoscopic screening were reconstructed using the population lists at the time of investigation and files of death certificates before the list was made in the study areas. Residence was limited to the same area in each city, because access to screening differed even for subjects who lived in the same city. Even if the subjects were matched, individuals under 40 years of age were excluded because they had no opportunity to be screened. Subjects who had gastric cancer when endoscopic screening was introduced were excluded and other control subjects were selected again based on the basic requirements. Finally, 146 case subjects and 794 matched control subjects in Tottori Prefecture and 264 case subjects and 1,498 matched control subjects in Niigata City were selected. Of the 410 case subjects, 343 had 6 control subjects, 22 had 5, 16 had 4, 12 had 3, 7 had 2, and 10 had 1.

### Screening History

The screening histories of the case and control subjects were obtained from the participant lists for both endoscopic and radiographic screenings for gastric cancer from April 2000 to March 2006 in Tottori Prefecture and from April 2003 to March 2010 in Niigata City. For some of the participants from Tottori and Kurayoshi, the method used for gastric cancer screening was unknown as some detailed information was lost. When the screening method was unclear, we assumed that there was no screening history. The end of the exposure period for screening was defined as the time when the case subjects were diagnosed as having gastric cancer.

### Statistical Analysis

Conditional logistic-regression models for matched sets were used to estimate the odds ratios (ORs) and 95% confidence intervals (95% CIs). The ORs were calculated for those who had participated in endoscopic or radiographic screening within 12, 24, 36, and 48 months before the reference date when the case subjects were diagnosed as having gastric cancer, compared with individuals who had never participated in any screening. The ORs were also calculated for those who had participated in each screening category within 36 months before the date of diagnosis, compared with individuals who had never participated in any screening by sex, 2 age groups of the case subjects (40–69 and 70–79 years), and 2 prefectures (Tottori and Niigata). Statistical analyses were carried out using STATA 11.0 (STATA, College Station, TX, USA).

This study used the data of local cancer screening programs and the population lists which were not included in the informed consents for the collection of the screening results and health data. Based on the Japanese guideline for epidemiological studies developed by the national government, informed consent is not required for an observational study using no human materials [14]. Since the design of our study was a case-control study, obtaining informed consent was waived. This study was approved by the Institutional Review Board of the National Cancer Center of Japan.

## Results

The total number of case subjects was 410, with 2,292 matched control subjects. The sex and age distributions of the case and control subjects are shown in **Table 1**. There were more men than women as case subjects (288 men, 122 women); 44% of the case subjects for both men and women were over 70 years of age.

**Table 1:** Distribution of case and control subjects by sex and age.

Age	Men				Women				Total			
	Cases	(%)	Control	(%)	Cases	(%)	Control	(%)	Cases	(%)	Control	(%)
40–49 years	11	3.8	57	3.6	12	9.8	63	9.2	23	5.6	120	5.2
50–59 years	39	13.5	242	15.1	23	18.9	125	18.2	62	15.1	367	16.0
60–69 years	111	38.5	597	37.2	35	28.7	198	28.8	146	35.6	795	34.7
70 years and over	127	44.1	708	44.1	52	42.6	302	43.9	179	43.7	1010	44.1
Total	288	100.0	1604	100.0	122	100.0	688	100.0	410	100.0	2292	100.0

The ORs were calculated for those who had participated in endoscopic or radiographic screening within 12, 24, 36, and 48 months before the reference date when the case subjects were diagnosed as having gastric cancer, compared with individuals who had never participated in any screening (**Table 2**). Compared with those who had never been screened before the date of diagnosis of gastric cancer in the case subjects, the ORs within 36 months from diagnosis were 0.695 (95% CI: 0.489–0.986) for endoscopic screening and 0.865 (95% CI: 0.631–1.185) for radiographic screening. The ORs of radiographic screening were not changed when the exposure window was changed from 12 months to 48 months. Although the results of radiographic screening suggested a reduction in mortality from gastric cancer, these were not significant. The OR within 12 months from diagnosis was 0.964 (95% CI: 0.660–1.407) for endoscopic screening. The ORs within 12 months were higher in endoscopic screenings than the ORs within 24, 36, and 48 months.

**Table 2:** Odds ratios of death from gastric cancer for screened subjects compared with never-screened subjects.

Months beforereference date	Number of subjects		Number of subjectsscreened by endoscopy				Odds ratio	Number of subjectsscreened by radiography				Odds ratio(95% CI)
	Cases	Controls	Cases	(%)	Controls	(%)	(95% CI)	Cases	(%)	Controls	(%)	
Within 12 months	410	2292	38	9.3	207	9.0	0.964	35	8.5	219	9.6	0.837
							(0.660–1.407)					(0.565–1.240)
24 months	410	2292	41	10.0	301	13.1	0.702	50	12.2	312	13.6	0.843
							(0.490–1.006)					(0.601–1.182)
36 months	407	2275	44	10.8	326	14.3	0.695	60	14.7	363	16.0	0.865
							(0.489–0.986)					(0.631–1.185)
48 months	387	2167	46	11.9	332	15.3	0.714	64	16.5	398	18.4	0.843
							(0.507–1.007)					(0.621–1.146)

The odds ratios were calculated for those who had participated in endoscopic or radiographic screening within 12, 24, 36, and 48 months before the reference date when the case subjects were diagnosed as having gastric cancer, compared with individuals who had never participated in any screening.

The ORs within 36 months by subgroups including sex, age group of the case subjects and prefecture are shown in **Table 3**. For men, the ORs were 0.560 (95% CI: 0.359–0.873) for endoscopic screening and 0.891 (95% CI: 0.611–1.229) for radiographic screening. The ORs for women were reversed between endoscopic screening and radiographic screening, but they were not significant. The ORs of endoscopic screening were 0.852 (95% CI: 0.504–1.440) in the 40–69 years age group and 0.593 (95% CI: 0.371–0.948) in the 70 years age group. The ORs of radiographic screening in both age groups were 1.015 (95% CI: 0.648–1.591) and 0.748 (95% CI: 0.483–1.161), but these were not significant. Although endoscopic screening was conducted in both prefectures, the ORs of both screening methods did not coincide. The ORs of both screenings were lower in Tottori Prefecture than in Niigata City. In 4 cities in Tottori Prefecture, the ORs were 0.451 (95% CI: 0.228–0.895) for endoscopic screening, and 0.498 (95% CI: 0.255–0.976) for radiographic screening.

**Table 3:** Odds ratios of death from gastric cancer for subjects screened compared with never-screened subjects within 36 months by sex, age group, and local area.

Subgroup	Monthsbefore diagnosis	Number of subjects		Number of subjects screenedby endoscopy				Odds ratio	Number of subjects screenedby radiography				Odds ratio
		Cases	Controls	Cases	(%)	Controls	(%)	(95% CI)	Cases	(%)	Controls	(%)	(95% CI)
Sex	Men	286	1593	26	9.1	236	14.8	0.560	42	14.7	241	15.1	0.891
								(0.359–0.873)					(0.611–1.299)
	Women	121	682	18	14.9	90	13.2	1.075	18	14.9	122	17.9	0.801
								(0.601–1.922)					(0.450–1.425)
Age group of case subjects	40–69 years old	229	1291	20	8.7	128	9.9	0.852	29	12.7	160	12.4	1.015
								(0.504–1.440)					(0.648–1.591)
	70 years old and over	178	984	24	13.5	198	20.1	0.593	31	17.4	203	20.6	0.748
								(0.371–0.948)					(0.483–1.161)
Prefecture	Niigata	264	1498	33	12.5	218	14.6	0.829	48	18.2	257	17.2	1.044
								(0.550–1.247)					(0.728–1.498)
	Tottori	143	777	11	7.7	108	13.9	0.451	12	8.4	106	13.6	0.498
								(0.228–0.895)					(0.255–0.976)

## Discussion

To the best of our knowledge, this is first case-control study of endoscopic screening for gastric cancer in communities in Japan. The results suggest a 30% reduction in gastric cancer mortality by endoscopic screening compared with no screening within 36 months before the date of diagnosis of gastric cancer. However, a case-control study may have potential bias, and care is needed when interpreting the results, because some serious biases may lead to a positive effect being found [13], [15]. Self-selection bias could not be controlled, and it may have also affected the results. Since the control subjects were screened more often than the case subjects, this will bias the results in favor of screening. To avoid this problem, a randomized controlled study to evaluate efficacy is required before introducing endoscopic screening in communities.

The ORs were higher in radiographic screening than in endoscopic screening even when the screening window was changed from 24 months to 48 months. Most of the case-control studies of radiographic screening suggested a significant decrease by 40–60% in gastric cancer mortality, and the results of previous studies were consistent [4], [16]–[20]. The effectiveness of radiographic screening was lower in the present study than in previous studies. The screening rate for gastric cancer using radiography has gradually decreased in the last decade and has remained at about 12% [5]. When the methods of gastric cancer screening were divided into endoscopy and radiography in study areas, the participation rate for each method was about 10% [7], [9]. Radiographic screening needs appropriate quality assurance for the radiographic technology and interpretation of the radiogram. Since most specialists who are in charge of this screening have become older, the quality assurance has become outdated. Because of our small sample size, the low screening rate, and insufficient quality assurance, significant results were difficult to obtain.

Sub-analysis was performed by dividing the subjects into 2 groups of age, sex and prefectures. Although significant results could not be obtained in the sub-analysis, different beneficial effects were found in these groups. Endoscopic screening has been mainly performed in clinical settings, and radiographic screening in mass screening programs in the study areas. Since over 70% of older people have their own family physician [21], they have more chance to be screened by endoscopy based on their physician’s recommendation. Therefore, the ORs of the subjects aged 70 years and over are lower than the ORs of the subjects aged 40 to 69 years. Although the basic screening programs were similar in Niigata and Tottori Prefectures, the quality assurance system is different [7], [9]. This has resulted in different beneficial effects. In the sub-analysis of sex, the ORs of radiographic screening are similar, but different beneficial effects were found in endoscopic screening. In the study areas, the screening method could be chosen based on individual preference. Preference of the screening method might be different between men and women.

This study had several limitations. Firstly, symptomatic individuals could not be excluded. The subjects of a case-control study to evaluate the effects of cancer screening should be asymptomatic [13], [22]. The ORs within 12 months were higher in endoscopic screenings than the ORs within 24, 36, and 48 months. The results suggest that symptomatic individuals might be screened more often within 12 months before diagnosis because of their symptoms. Symptomatic individuals have often participated in cancer screening instead of diagnostic tests, and free-of-charge programs for the elderly people in these cities have promoted examinations of symptomatic individuals.

Secondly, since the background information, including smoking and family history, was not obtained, no adjustments could be made for the differences. Fukao et al. reported differences in family history and smoking between participants and non-participants in gastric cancer screening [23]. Lifestyle might differ between the subjects who participate or not in screening. Since half of the case and control subjects was 70 years old and over, they had a high potential to have co-morbidity. The different results for endoscopic screening among the older and younger age groups might be affected by screening history and co-morbidities.

Thirdly, the screening history outside of community-based screening was unclear. Screening history was identified based on the participant lists for gastric cancer screening from 2000 to 2006. Opportunistic screening was popular in clinical settings, mainly using endoscopy. In addition, in the 40–59 years age group, gastric cancer screening was often performed with regular health check-ups in the workplace [24].

Lastly, since the new screening method had not been well known in the community at the time when endoscopic screening was first introduced, the chance to be screened might have been missed. Since there is no existing system for inviting the target population to participate in cancer screening in most municipalities in Japan, with gastric cancer screening offered annually, most subjects themselves elected to participate irregularly. The minimum period for selection was 3 years after the introduction of endoscopic screening. There is an argument that one should define the available time for the determination of case subjects to assess the effectiveness of screening accurately [13]. A screening effect is not expected within a short time after introduction; it needs several years [25]. Wahrendof et al. defined the cases as deaths from colorectal cancer occurring more than 6 years after the introduction of screening that was fully implemented [26]. Although there was an equal chance to participate in both screening modalities, individuals who continued to be screened tended to change to endoscopy. If the long-term effect of radiographic screening continued, the effectiveness of endoscopic screening might be overestimated.

In conclusion, our results suggest a 30% reduction in mortality from gastric cancer by endoscopic screening within 36 months from the diagnosis of gastric cancer in case subjects compared with never-screened subjects. Although this suggests the effectiveness of endoscopic screening for gastric cancer, several limitations, including self-selection bias, remain, and prudent interpretation is needed. A randomized controlled study to evaluate efficacy is required before introducing endoscopic screening in communities.
